# Supplementary material for: Asynchronous magnetic resonance elastography: Shear wave speed reconstruction using noise correlation of incoherent waves
Source: Magn Reson Med. 2022 Oct 27;89(3):990–1001. doi: 10.1002/mrm.29502 (PMC9792433; doi:10.1002/mrm.29502)
Supplement: Supplementary file 1 — DATA S1 MATLAB code used to generate the numerical simulations of Figure 1 [file MRM-89-990-s002.zip › k-Wave/helpfiles/example_us_defining_transducer.html]

Defining An Ultrasound Transducer Example (k-Wave)


# Defining An Ultrasound Transducer Example

In principle, simulations using diagnostic ultrasound transducers can be run following the examples given under Time Varying Source Problems. However, assigning the grid points that belong to each transducer element, and then assigning the correctly delayed input signals to each point of each element can be a laborious task. For this purpose, a special input object created using the `kWaveTransducer` class can be substituted for either the `source` or `sensor` inputs (or both). This example illustrates how this object is created and can be used to simulate the field produced by an ultrasound transducer.

- Open the file in the MATLAB Editor
- Run the file in MATLAB

Note, transducer inputs can only be used in 3D simulations and thus these examples are inherently memory and CPU intensive. Whilst the grid sizes and source frequencies used in the examples have been scaled down for the purpose of demonstrating the capabilities of the toolbox (the inputs do not necessarily represent realistic ultrasound settings), they still require a comparatively large amount of computational resources. To reduce this load, it is advised to run the simulations in single precision by setting the optional input `'DataCast'` to `'single'`. Similarly, if you have access to a recent model GPU and the MATLAB Parallel Computing Toolbox R2012a or later, the simulation times can be significantly reduced by setting `'DataCast'` to `'gpuArray-single'`. Alternatively, the simulations can be run using the optimised C++ code. See the k-Wave Manual for more information.

The creation of a `kWaveTransducer` object will only work in versions of MATLAB recent enough to support user defined classes.

## Contents

- Defining the input signal
- Defining the k-Wave Transducer
- Running the simulation

## Defining the input signal

If the transducer is to be used as an ultrasound source, the input signal used to drive the transducer elements must be defined before the transducer is created. A single input signal is used to drive the transducer, with the beamforming delays later calculated automatically depending on the user settings for the transducer focus distance and steering angle. The input signal can be any 1D vector. Here, this is created using `toneBurst`. This creates a single frequency sinusoid with a given number of cycles windowed by a Gaussian. Note, the properties of the input signal are dependent on the time step used in the simulation, so `kgrid.t_array` must be defined before creating the input signal.

```
% define properties of the input signal
source_strength = 1e6;          % [MPa]
tone_burst_freq = 0.5e6;        % [Hz]
tone_burst_cycles = 5;

% create the input signal using toneBurst
input_signal = toneBurst(1/kgrid.dt, tone_burst_freq, tone_burst_cycles);
```

In the current version of k-Wave, only linear (flat) transducers are supported. In this case, the input signal is assigned to the particle velocity in the direction the transducer is facing (`source.ux` if the input was being assigned manually). Consequently, the input signal must be scaled to be in units of velocity rather than pressure.

```
% scale the source magnitude by the source_strength divided by the
% impedance (the source is assigned to the particle velocity)
input_signal = (source_strength ./ (medium.sound_speed * medium.density)) .* input_signal;
```

A plot of the resulting input signal and its frequency spectrum is given below. The more cycles in the tone burst, the narrower the frequency spectrum becomes about the central frequency.

Because shifted (delayed) versions of the input signal are used to control the directionality and focus of the signal from the transducer, extra zeros must be appended to the start and end of the input signal. The number of zeros is checked by k-Wave and additional zeros are prepended and appended automatically if required. It is also possible to fix the number of added zeros by defining `transducer.steering_angle_max`. In this case the number of added zeros is calculated based on the maximum steering angle. This behaviour is useful if forming an ultrasound image by steering the beam through a range of angles.

## Defining the k-Wave transducer

After the input signal has been created, the transducer can then be defined. This is accomplished by creating an object of the `kWaveTransducer` class. The characteristics of the transducer are dependent on a number of properties, some of which are fixed when the transducer is created (for example the position of the transducer and the number of elements) and some of which can be defined or modified at any time (for example the steering angle or focus distance). Because of the large number of input combinations, the user defined input properties are appended as fields to an input structure. This structure can be given any name. In this example, both the input structure and the created `kWaveTransducer` object are called `transducer`.

First, the physical properties of the transducer are defined, including the number of elements and their size. The sizes are all given in units of grid points which means the physical size is also dependent on the values of `kgrid.dx`, `kgrid.dy`, and `kgrid.dz`.

```
% physical properties of the transducer
transducer.number_elements = 72;    % total number of transducer elements
transducer.element_width = 1;       % width of each element [grid points]
transducer.element_length = 12;     % length of each element [grid points]
transducer.element_spacing = 0;     % spacing (kerf width) between the elements [grid points]
transducer.radius = inf;            % radius of curvature of the transducer [m]
```

The `radius` input will be used in future versions of k-Wave to create curved transducers. In the current version, only linear (flat) transducers are supported, so this variable must be set to `inf` or not defined (this will default to `inf`). A schematic illustrating some of the common terminology used to describe the physical properties of ultrasound transducers is given below.

The transducer is defined within the computational grid such that the front face of the transducer is pointing towards the positive x-direction. The position of the transducer within the grid is set using the `position` field which defines the position of the nearest grid point belonging to the transducer relative to the grid origin. For example, if this is set to `(1, 1, 1)`, the corner of the transducer will be positioned flush with the grid origin. In the current example, the position is defined such that the transducer is centered within the computational grid.

```
% calculate the width of the transducer in grid points
transducer_width = transducer.number_elements * transducer.element_width ...
    + (transducer.number_elements - 1) * transducer.element_spacing;

% use this to position the transducer in the middle of the computational grid
transducer.position = round([1, Ny/2 - transducer_width/2, Nz/2 - transducer.element_length/2]);
```

Next, the dynamic properties of the transducer are defined. These control the characteristics of the transducer output and can be modified at any stage after the transducer has been created. If any of these parameters are not defined by the user, they are given default values (see `kWaveTransducer` for a list of these defaults). The sound speed, focus distance, and steering angle are used to calculate the beamforming delays based on standard geometric expressions. The elevation focus distance is used to mimic the focussing behaviour of real transducers in which an acoustic lens is used to focus the beam in the out of plane (x-z) or elevation direction. Within k-Wave, this behaviour is modelled by using an additional set of beamforming delays across the grid points within each element.

```
% properties used to derive the beamforming delays
transducer.sound_speed = 1540;                  % sound speed [m/s]
transducer.focus_distance = 20e-3;              % focus distance [m]
transducer.elevation_focus_distance = 19e-3;    % focus distance in the elevation plane [m]
transducer.steering_angle = 0;                  % steering angle [degrees]
```

The apodization (this controls the relative weights assigned to the signals driving each of the active transducer elements) is defined as a string corresponding to any valid window type supported by `getWin`. It can also be manually defined as a 1D vector of relative weights applied to the active transducer elements, or not defined (this defaults to `'Rectangular'`). The transmit apodization is applied when the transducer is used as a source, while the receive apodization is applied when the transducer is used as a sensor (see Using An Ultrasound Transducer As A Sensor Example).

```
% apodization
transducer.transmit_apodization = 'Rectangular';    
transducer.receive_apodization = 'Rectangular';
```

While typical diagnostic ultrasound transducers may have 128 transducer elements, frequently only a small subset of these are used to transmit and receive ultrasound signals at any particular time. The transducer elements that are currently active can be defined using the `active_elements` field which is assigned as a 1D binary mask. In this example, the central 32 elements are set to be active.

```
% define the transducer elements that are currently active
transducer.active_elements = zeros(transducer.number_elements, 1);
transducer.active_elements(21:52) = 1;
```

Finally, the input signal defined in the previous section is also added to the input structure, and the transducer is created using `kWaveTransducer`. This returns an object of the `kWaveTransducer` class.

```
% append input signal used to drive the transducer
transducer.input_signal = input_signal;

% create the transducer using the defined settings
transducer = kWaveTransducer(kgrid, transducer);
```

Note, in this example, both the input structure and the output object are given the same name. While they share many of the same properties, these are not the same (the former is overwritten by the latter in the final line above). The input is just a holder for all the properties that have been defined, while the output is an object of the `kWaveTransducer` class which has additional properties and methods. As an example, a summary of the properties of the transducer can be printed to the command line by calling the method `transducer.properties`. A voxel plot of the transducer can also be automatically generated by calling `transducer.plot`. A plot of the transducer created in the current example is given below.


### Running the simulation

Once the transducer has been defined, the simulation is run by calling `kspaceFirstOrder3D`. In this example, the transducer is used to replace the `source` input, with the remaining inputs defined in the normal fashion. Here, a homogeneous medium is defined and the `sensor` input is given as a binary mask with three sensor positions along the transducer axis. Because the amplitude of the ultrasound waves generated by most ultrasound transducers is sufficiently high to generate nonlinear effects, a value for `medium.BonA` (the parameter of nonlinearity) should also be set. When this value is defined, k-Wave includes nonlinear effects in the governing equations. A snapshot of the simulation is given below.

```
% run the simulation
[sensor_data] = kspaceFirstOrder3D(kgrid, medium, transducer, sensor, input_args{:});
```

The recorded sensor data is returned as normal. A plot of the recorded time series along with their amplitude spectrums is given below. Notice some of the energy at the source frequency has been transferred to the second harmonic due to nonlinear effects. In practice, the generation of higher frequency harmonics via nonlinearity is delicately balanced with their absorption, and thus the shape of the simulated waveforms will be dependent on the values set for these parameters.
